# The Lysozyme Inhibitor Thionine Acetate Is Also an Inhibitor of the Soluble Lytic Transglycosylase Slt35 from *Escherichia coli*

**DOI:** 10.3390/molecules26144189

**Published:** 2021-07-09

**Authors:** Aysha B. Mezoughi, Chiara M. Costanzo, Gregor M. Parker, Enas M. Behiry, Alan Scott, Andrew C. Wood, Sarah E. Adams, Richard B. Sessions, E. Joel Loveridge

**Affiliations:** 1 School of Chemistry, Cardiff University, Park Place, Cardiff CF10 3AT, UK; abamezoughi2013@gmail.com (A.B.M.); gregor.m.parker@gmail.com (G.M.P.); behiryem@cardiff.ac.uk (E.M.B.); scotta4@cardiff.ac.uk (A.S.); andrew.wood@catsci.com (A.C.W.); sarah.adams@evotec.com (S.E.A.); 2 Department of Chemistry, Swansea University, Singleton Park, Swansea SA2 8PP, UK; 993910@swansea.ac.uk; 3 School of Biochemistry, University of Bristol, University Walk, Bristol BS8 1TD, UK; r.sessions@bristol.ac.uk

**Keywords:** lytic transglycosylase, thionine acetate, enzyme inhibition, antibacterial

## Abstract

Lytic transglycosylases such as Slt35 from *E. coli* are enzymes involved in bacterial cell wall remodelling and recycling, which represent potential targets for novel antibacterial agents. Here, we investigated a series of known glycosidase inhibitors for their ability to inhibit Slt35. While glycosidase inhibitors such as 1-deoxynojirimycin, castanospermine, thiamet G and miglitol had no effect, the phenothiazinium dye thionine acetate was found to be a weak inhibitor. IC_50_ values and binding constants for thionine acetate were similar for Slt35 and the hen egg white lysozyme. Molecular docking simulations suggest that thionine binds to the active site of both Slt35 and lysozyme, although it does not make direct interactions with the side-chain of the catalytic Asp and Glu residues as might be expected based on other inhibitors. Thionine acetate also increased the potency of the beta-lactam antibiotic ampicillin against a laboratory strain of *E. coli*.

## 1. Introduction

Bacterial cells are surrounded by a peptidoglycan sacculus on the outside of the cytoplasmic membrane, which is essential for maintaining cell strength and integrity [1]. Peptidoglycan is composed of glycan strands of repeating *N*-acetylglucosamine (NAG) and *N*-acetylmuramic acid (NAM) residues linked by β-1,4-glycosidic bonds, cross-linked via peptide side-chains [2]. *Escherichia coli* turns over about 50% of its sacculus per generation, to allow cell growth and division, insertion of proteins into the cell wall and other processes [3]. The muropeptides released from the sacculus are recycled by the cell. The sacculus degradation and remodelling activities are catalysed by enzymes including glycosidases, amidases, endopeptidases and carboxypeptidases [4,5].

Lytic transglycosylases are important peptidoglycan-degrading glycosidases [6,7,8], that catalyse cleavage of the β-1,4-glycosidic bond between NAM and NAG residues. Unlike lysozymes, they do so by catalysing an intramolecular transglycosylation reaction that forms 1,6-anhydromuropeptides [9], which can be recycled to form fresh peptidoglycan but which may also act as inducers of β-lactamase production [10] and as virulence factors [11,12,13]. Many bacterial species, including human pathogens such as *Bacillus anthracis, Staphylococcus aureus, Neisseria meningitides* and *Neisseria gonorrhoeae*, inhibit the activity of both lysozyme and lytic transglycosylases by acetylation of C-6 hydroxyl moieties of NAM residues in their peptidoglycan [14,15]. This is used to control lytic transglycosylase activity and so prevent autolysis [7]. Vertebrate lysozymes are also inhibited by the proteinaceous inhibitor Ivy, which is produced by certain Gram-negative bacteria [16]. Inhibition of the membrane-bound lytic transglycosylase MltB from *P. aeruginosa* by Ivy was suggested to regulate the autolytic activity of lytic transglycosylases in Gram-negative bacteria that are unable to O-acetylate their peptidoglycan [17]. However, despite preventing autolysis, specific inhibition of LTs may also increase the potency of β-lactam antibiotics against bacteria, as exemplified by the action of the bulgecins [18,19,20,21,22,23,24,25].

**Figure 1:**
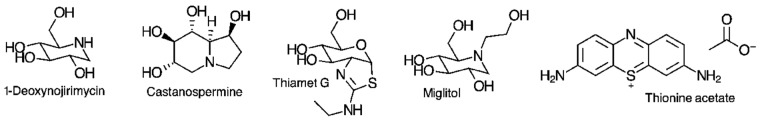
Structures of some reported glycosidase inhibitors and thionine acetate.

Iminosugars, alkaloids and their synthetic analogues (Figure 1) can act as glycosidase inhibitors by mimicking the glycosyloxocarbenium ion intermediate and related transition states in enzymatic glycoside hydrolysis [26]. 1-Deoxynojirimycin, a D-glucose analogue, is the best known iminosugar. The alkaloid castanospermine is also a β-glucosidase inhibitor [27]. The NAG-thiazoline derivative thiamet G is a potent inhibitor of β-acetylglucosaminidases [28], while miglitol (Glyset) is used as a drug for treatment of type II diabetes [29]. Thionine acetate, a planar cationic phenothiazinium dye, has been shown to bind to lysozyme [30]. This compound is also used in electrochemical and photochemical biosensors [31,32] and has antibacterial activity toward pathogenic bacteria [33].

In this study, we evaluated thionine acetate and some known glycosidase inhibitors against hen egg white lysozyme and Slt35, using a combination of enzyme activity assays, dissociation constant determination and molecular docking analysis. 

## 2. Results and Discussion

### 2.1. Enzyme Activity Assay

#### 2.1.1. Effect of the Buffer

A turbidimetric assay [34] was used to determine the activity of hen egg white lysozyme and Slt35, by monitoring peptidoglycan solubilisation when the enzyme is added to *Micrococcus lysodeikticus* cell suspension. Although this assay has previously been performed in sodium phosphate buffer for the same enzymes [17], we found Tris-maleate to give superior enzyme activity: maximum 0.018 A min^−1^ for phosphate (pH 6.4) and 0.068 A min^−1^ for Tris-maleate (pH 5.8), both containing 100 mM NaCl. Acetate, MES and HEPES buffers gave very poor activity: maximum 0.020 A min^−1^ for sodium acetate (pH 5.5), 0.003 A min^−1^ for MES (pH 6.0) and 0.003 A min^−1^ for HEPES (pH 7.0), all containing 100 mM NaCl. 

#### 2.1.2. Effect of Salts

**Figure 2:**
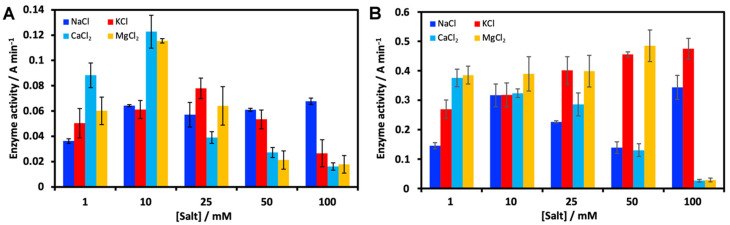
Effect of salt concentration on (**A**) Slt35 and (**B**) lysozyme activity. For Slt35, 7 μM enzyme and 0.6 mg ml^−1^ cell suspension were used at 37 °C in 25 mM Tris-maleate (pH 5.8). For lysozyme, 0.1 μM enzyme and 0.6 mg ml^−1^ cell suspension were used at 37 °C in 25 mM Tris-maleate (pH 6.0).

The effect of NaCl, KCl, MgCl_2_ and CaCl_2_ on enzyme activity was determined. The activity of Slt35 was not determined in the absence of salts, due to very low activities and difficulty in handling and storing the protein. Monovalent cations did not have a significant effect on Slt35 activity, whereas 10 mM divalent cations gave optimal activity (Figure 2A). The thermostability of Slt35 has been shown to increase when it is bound to calcium ions, but not sodium, potassium or magnesium ions at 10 mM [35]. The melting temperature of Slt35 increased from 48.0 °C in the absence of salts to 55.5 °C in the presence of 1 mM CaCl_2_, suggesting that the EF-hand calcium binding site of Slt35 plays an important role in protein stability [35]. However, the functional role of this site has not been established. The effect of salts on lysozyme activity was also assessed (Figure 2B). Potassium was found to be the optimal cation in this case, as other cations caused a reduction in lysozyme activity at higher concentrations.

#### 2.1.3. Effect of pH

**Figure 3:**
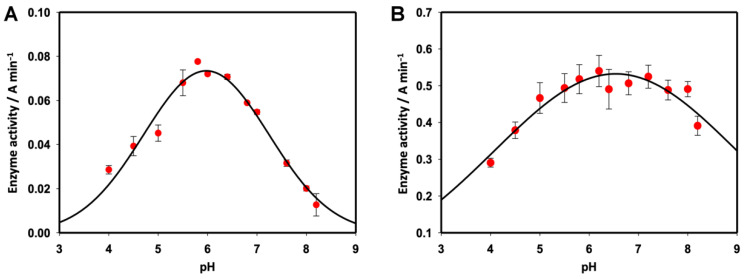
Effect of pH on (**A**) Slt35 and (**B**) lysozyme activity. For Slt35, 7 μM enzyme and 0.6 mg mL^−1^ cell suspension were used at 37 °C in 25 mM Tris-maleate containing 10 mM CaCl_2_. For the lysozyme, 0.1 μM enzyme and 0.6 mg mL^−1^ cell suspension were used at 37 °C in 25 mM Tris-maleate containing 100 mM KCl.

The activity of Slt35 showed a bell-shaped dependence on pH, with maximum activity at pH 5.8 (Figure 3A). Lysozyme displayed a much broader pH range giving maximum activity, and was generally more tolerant of changes in the pH, although activity was decreased below pH 5 and above pH 8 (Figure 3B). It has been noted that the pH dependence of lysozyme activity is strongly affected by the ionic strength [36], which was different for the two enzymes here. The accepted mechanism of catalysis [37] by the hen egg white lysozyme requires Asp52 to be deprotonated and Glu35 to be protonated. The p*K*_a_ values of these residues are 3.7 and 6.2 respectively [38], accounting for the reduction in activity at low and high pH. The suggested mechanism for Slt35 requires Glu162 to be protonated, but there is no requirement for an equivalent deprotonated residue. The side-chain p*K*_a_ of free glutamate is 4.5, suggesting that Glu162 has its p*K*_a_ value perturbed to a higher value by its local environment, as seen in the hen egg white lysozyme [38] and many other enzymes [39]. The loss of activity at lower pH is then likely to be due to unfavourable protonation of a different residue, with a p*K*_a_ below 6, that may affect substrate binding or the overall arrangement of the active site.

#### 2.1.4. Effect of the Substrate

**Figure 4:**
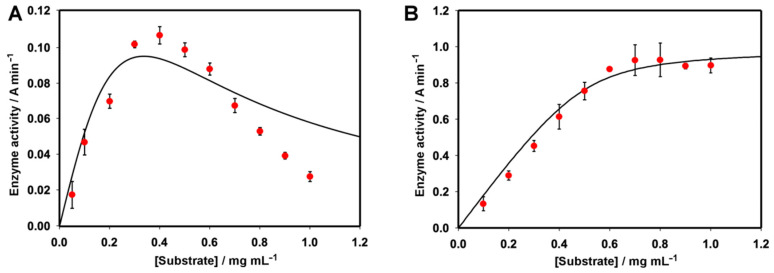
(**A**): Effect of the substrate concentration on (**A**) Slt35 and (**B**) lysozyme activity. For Slt35, the 7 μM enzyme was used at 37 °C in 25 mM Tris-maleate (pH 5.8) containing 10 mM CaCl_2_. A fit to an expression for substrate inhibition [40] is shown. For lysozyme, 0.1 μM enzyme was used at 37 °C in 25 mM Tris-maleate (pH 6.0) containing 100 mM KCl. A fit to the Morrison equation for the tight-binding substrate [41] is shown.

Slt35 showed maximum activity at a substrate concentration of 0.4 mg mL^−1^ (Figure 4A), in keeping with previous studies, where substrate concentrations of 0.4–0.6 mg mL^−1^ were used [17]. Above this, Slt35 activity reduced significantly, demonstrating substrate inhibition [40]. The optimal lysozyme activity was observed at substrate concentrations above 0.6 mg/mL (Figure 4B). Unlike Slt35, the lysozyme was not inhibited by further increases in the substrate concentration, in the range used here. Due to the high concentration of the enzyme used here, lysozyme did not follow Michaelis–Menten kinetics, so the data were instead fit to the Morrison equation for tight binding of the substrate [41], giving an apparent *K*_m_ of 0.034 mg mL^−1^ cell suspension. This is in keeping with previous studies, which found *K*_m_ values in the region of 0.01–0.05 mg mL^−1^ for *M. lysodeikticus* cell suspension [42], and a *K*_d_ value of 14 μM for chitotriose [43]. As Slt35 displayed both substrate inhibition and tight substrate binding, we were unable to obtain good fits for the experimental data. Fitting to an expression for substrate inhibition based on Michaelis–Menten kinetics [40] gave a value of 7.54 mg mL^−1^ for *K*_m_ and 0.0012 mg mL^−1^ for *K*_I_. 

#### 2.1.5. Effect of Temperature

**Figure 5:**
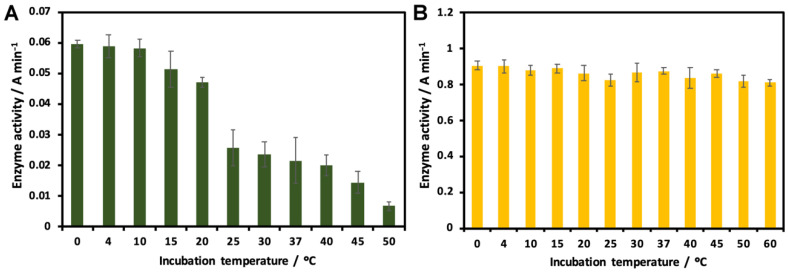
Effect of temperature on (**A**) Slt35 and (**B**) lysozyme activity. For Slt35, the 7 μM enzyme and 0.6 mg mL^−1^ cell suspension were used in 25 mM Tris-maleate (pH 5.8) containing 10 mM CaCl_2_. For the lysozyme, the 0.1 μM enzyme and 0.6 mg mL^−1^ cell suspension were used in 25 mM Tris-maleate (pH 6.0) containing 100 mM KCl. The enzyme was incubated for 10 min at the temperatures indicated before being added to the substrate. Activity was monitored at 37 °C.

To investigate the effect of temperature on enzymatic activity, Slt35 and the lysozyme were incubated at different temperatures for 10 min before they were added to the substrate. The maximum lytic activity of Slt35 was recorded when the enzyme was incubated below 15 °C, while the enzyme lost about half of its activity at 25–40 °C, and at 50 °C the enzyme precipitated (Figure 5A). Increasing the incubation temperature as high as 60 °C did not show any significant effect on lysozyme activity (Figure 5B). Although all kinetic measurements in this work were performed at the physiologically relevant temperature of 37 °C, the enzyme was always stored at <4 °C, and all incubations with potential inhibitors were performed on ice to avoid denaturation of the enzyme prior to measurements. We did not expect significant denaturation of the enzyme to occur during the 1 min assay at 37 °C; initial rates were linear. Others have also performed experiments with Slt35 at temperatures of 25–37 °C [18,44,45]. 

### 2.2. Enzyme Inhibition Studies

**Figure 6:**
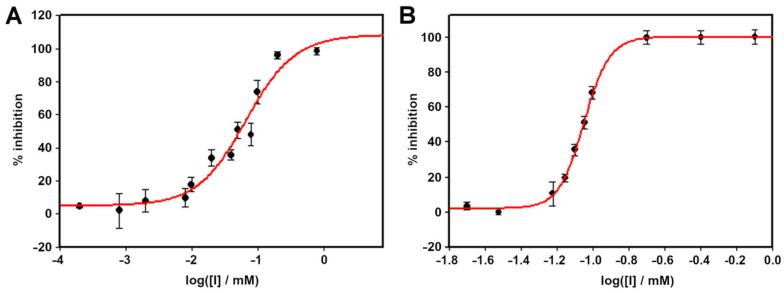
Inhibition of (**A**) Slt35 and (**B**) lysozyme by thionine acetate. For Slt35, 7 μM enzyme and 0.6 mg mL^−1^ cell suspension were used at 37 °C in 25 mM Tris-maleate buffer (pH 5.8) containing 10 mM CaCl_2_. For lysozyme, 0.1 μM enzyme and 0.6 mg mL^−1^ cell suspension were used at 37 °C in 25 mM Tris-maleate buffer (pH 6.0) containing 100 mM KCl.

The same turbidimetric assay was used to evaluate the inhibition of lysozyme and Slt35. 1-Deoxynojirimycin, castanospermine, thiamet G and miglitol did not inhibit either enzyme at concentrations up to 10 mM. Thionine acetate was also evaluated for inhibition of Slt35 and HEWL. Increasing the concentration of thionine acetate reduced the rate of the lytic reaction for both enzymes (Figure 6). The IC_50_ was found to be 89.3 ± 3 µM for lysozyme and 66.0 ± 0.1 µM for Slt35.

### 2.3. Binding Studies

Interestingly, although 1-deoxynojirimycin did not inhibit Slt35, the saturation transfer difference NMR spectroscopy showed weak enhancement of signals from 1-deoxynojirimycin on selective excitation of Slt35 (Figure S1), suggesting that this compound is capable of binding to Slt35. Conversely, saturation transfer difference NMR did not detect any binding of castanospermine to Slt35.

**Figure 7:**
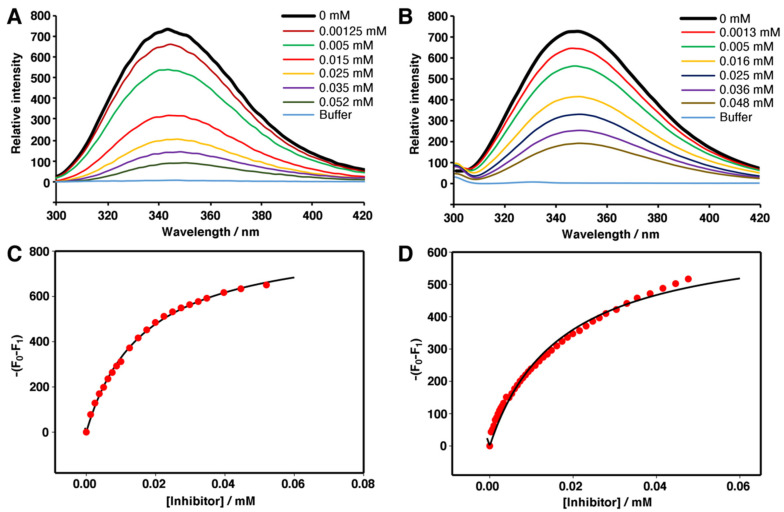
Fluorimetric titration of lysozyme or Slt35 with thionine acetate. Fluorescence emission of (**A**) 3 µM Slt35 and (**B**) 2.3 µM lysozyme in the presence of varying concentrations (indicated) of thionine acetate. Change in maximum fluorescence intensity of (**C**) lysozyme and (**D**) Slt35 with increasing thionine acetate concentration.

The extinction of the intrinsic fluorescence of Slt35 and lysozyme in the presence of thionine acetate was used to determine the binding constant. The effect of thionine acetate on the fluorescence emission of Slt35 and lysozyme was similar (Figure 7), and the *K*_d_ values of thionine acetate complexed to Slt35 and lysozyme were found to be 17.1 ± 1.0 and 18.6 ± 1.2 μM respectively.

### 2.4. Molecular Docking Studies

**Figure 8:**
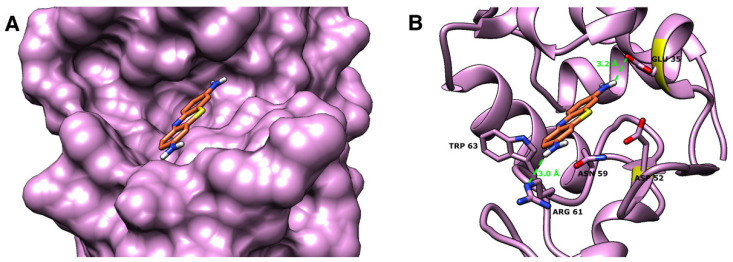
The predicted binding mode of thionine acetate with the hen egg white lysozyme (PDB 2VB1 [47]). The enzyme is shown in pink as (**A**) a surface representation of the binding site and (**B**) a cartoon representation showing the amino acid residues (as sticks) located in the active site close to the inhibitor. The inhibitor is shown as orange sticks. The predicted hydrogen bonding interactions are shown as green dashes with distances in Å.

Docking was performed using the Bristol University Docking Engine (BUDE) [46], to elucidate the binding mode of thionine acetate in the active of both Slt35 and hen egg white lysozyme. The docking suggests that thionine binding to lysozyme is dominated by H-bonding interactions between amino groups in the inhibitor and Glu35 and Arg61 residues in the lysozyme active site (Figure 8). Another, weaker, interaction could occur between Asn59 and the inhibitor.

The lysozyme–thionine binding mode obtained here is similar, but not identical, to that obtained in previous docking studies using the lower-resolution PDB 6LYZ [30], which showed thionine acetate forming hydrogen bonds to the main-chain carbonyl groups of Leu56 and Ile58 of lysozyme. In our work, thionine acetate is shifted more into the centre of the active site; the orientation and approximate position of thionine is similar to that observed in the previous work, but hydrogen bonds are made to different residues (Figure S2). To investigate whether the differences in the predicted binding mode are due to the different docking software used, we repeated all docking using AutoDock [48]. We found only small differences between the binding mode of thionine acetate predicted by BUDE or AutoDock when PDB 2VBI was used (Figure S3), and obtained poses very similar to those observed previously [30] when PDB 6LYZ was used (Figure S4). This suggests that the small differences in the lysozyme-thionine binding mode observed between our work and the previous studies are due to the different crystal structures used.

**Figure 9:**
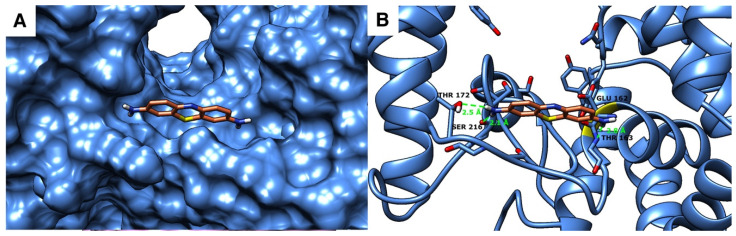
Predicted binding mode of thionine acetate with Slt35 (PDB 1QUS [49]). The enzyme is shown in blue as (**A**) a surface representation of the binding site and (**B**) a cartoon representation showing the amino acid residues (as sticks) located in the active site close to the inhibitor. The inhibitor is shown as orange sticks. The predicted hydrogen bonding interactions are shown as green dashes with distances in Å.

Docking of thionine with Slt35 suggested the presence of hydrogen bonds between the two amino groups of the inhibitor and the carbonyls of Glu162 and Gly215 residues (Figure 9). Another hydrogen-bonding interaction was observed between one of the amino groups in thionine and the side-chain hydroxyl of Thr172 in the active site of Slt35, while the side-chain of Val168 formed a hydrophobic interaction with one of the aromatic rings in thionine. As observed for lysozyme, the Slt35-thionine docking result was very similar when AutoDock was used (Figure S5).

Thionine does not make direct interactions with the side-chain of the key catalytic Glu162 of Slt35 and adopts a quite different binding mode to bulgecin A (Figure S6) [50]. Based on the interactions of bulgecin A with Slt35, an interaction between the cationic nitrogen of the inhibitor and the carboxylate side chain would be expected, to mimic the interaction between the carboxylate of Glu162 and the oxocarbenium ion intermediate [49,50]. However, such an interaction is not required for inhibition, as long as the active site is obstructed. Indeed, thionine sits across the mouth of the Slt35 active site (Figure 9, Figure S6), blocking binding of the large peptidoglycan chain. As the structure of thionine is quite different to that of peptidoglycan, and the active site of Slt35 contains numerous other residues capable of interacting with a cation [49,50], the different orientation of the inhibitor is not surprising.

Our docking studies suggest that the binding affinity of Slt35 for thionine could be improved by extending the thionine core at positions C3 and C13 (i.e., the carbon atoms between the amino groups and the ring bridgeheads; Figure 1). Extension at these positions with chains or rings containing polar groups would allow additional hydrogen bonding and/or ion-pair interactions within the active site, increasing affinity and boosting the inhibition of the enzyme.

### 2.5. Antibacterial Testing

Thionine acetate showed no antibacterial activity against *Escherichia coli* JM109 by disk diffusion assays. However, when inoculated onto the same disk as ampicillin, thionine acetate did give a small increase in the inhibition zone diameter (Table 1), suggesting synergism with the antibiotic. This is consistent with previous studies, where the lytic transglycosylase inhibitor bulgecin was shown to greatly increase the potency of beta-lactam antibiotics [18,19,22], and further supports the view that lytic transglycosylase inhibitors may be used clinically as combination therapies with beta-lactam antibiotics [7,8,51,52]. Bulgecin A is also an inhibitor of metallo-β-lactamases [53].

## 3. Materials and Methods

### 3.1. Chemical Reagents and Enzymes

A pET28a plasmid harbouring the gene encoding Slt35 with an N-terminal His-tag, between *Nde*I and *Bam*H1 restriction sites, was purchased from Epoch Life Science (USA). The hen egg white lysozyme was purchased from Sigma-Aldrich. Thionine acetate and other chemicals were purchased from Sigma-Aldrich or Fisher Scientific unless otherwise stated. 

### 3.2. Protein Production and Purification

*Escherichia coli* XL-1 Blue and BL21(DE3) cells were transformed with a pET28a plasmid harbouring the *slt35* gene using standard techniques. A single colony of freshly-transformed *E. coli* BL21(DE3) cells was inoculated in LB medium containing kanamycin (50 μg mL^−1^) and grown overnight at 37 °C and 180 rpm. This overnight culture was used to seed (1:50 dilution) fresh kanamycin-selective LB medium and growth was continued at 37 °C and 180 rpm until the OD_600_ was 0.6–0.8. *slt35* expression was induced using 120 mg L^−1^ isopropyl-β-D-thiogalactopyranoside and cultures were incubated at 18 °C overnight. The cells were harvested via centrifugation in a Sorvall RC 6 Plus centrifuge (Thermo Fisher Scientific, Inc, MA, USA) at 6080× *g*, and stored at –20 °C. When required, cells were resuspended in a minimal volume of 50 mM Tris-HCl buffer at pH 7.0 supplemented with 500 mM NaCl, 10% glycerol and 0.05% Tween-20, thawed and lysed by sonication on ice. Cell debris was removed by centrifuging at 17,065× *g* in a Sorvall RC 6 Plus centrifuge (Thermo Fisher Scientific, Inc, MA, USA). The supernatant solution was loaded onto a HiTrap Chelating column (Ni-NTA resin), which was then washed with the same buffer and bound protein eluted using a gradient of 0–500 mM imidazole in the same buffer. Fractions containing pure Slt35 were combined and dialysed overnight against 50 mM Tris-HCl buffer at pH 7.0 supplemented with 300 mM NaCl, 10% glycerol and 0.05% Tween-20. 

### 3.3. Enzyme Activity

Whole *Micrococcus lysodeikticus* cells were suspended in assay buffer and incubated at 37 °C before adding the purified enzyme (final concentration 0.1 μM for lysozyme and 7 μM for Slt35). The decrease in turbidity was monitored at 600 nm for 1 min at 37 °C, and the initial rate determined. A range of different buffers, pH, salt concentrations, substrate concentrations and temperatures were investigated. All investigations used three independent repeats of each condition, and results are expressed as the average ± standard deviation. The optimum assay buffer was found to be 25 mM Tris-maleate (pH 5.8) containing 10 mM CaCl_2_ for Slt35 and 25 mM Tris-maleate (pH 6.0) containing 100 mM KCl for lysozyme. A 0.6 mg mL^−1^ cell suspension was used in all subsequent assays. For the substrate dependence of the enzyme activity, the data for the lysozyme were fitted by nonlinear regression using SigmaPlot 10 to the Morrison equation for tight binding of the substrate [41]:

(1) $$v=v_{max}\frac{\left(E_{T}+S_{T}+K_{m}\right)-\sqrt{{\left(E_{T}+S_{T}+K_{m}\right)}^{2}-4E_{T}S_{T}}}{2E_{T}}$$

The data for Slt35 were fitted by nonlinear regression using SigmaPlot 10 to an expression for substrate inhibition based on Michaelis–Menten kinetics [40]:

(2) $$v=\frac{v_{max}S}{K_{m}+S+\frac{S^{2}}{K_{I}}}$$

### 3.4. Enzyme Inhibition

Slt35 or lysozyme was preincubated with the potential inhibitor for 10 min at 0 °C before being added to the assay mixture as above (Section 3.3). Three independent repeats were performed for each inhibitor. Initial rate data were normalised between the rate in the absence of the inhibitor and the rate in the absence of the enzyme, and reported as a percent of inhibition (where the normalised rate in the absence of the inhibitor = 0% inhibition and normalised rate in the absence of the enzyme = 100% inhibition). The data were fitted by nonlinear regression using SigmaPlot 10 (Ligand Binding, sigmoidal dose-response, variable slope):

(3) $$\%inhibition=min+\frac{max-min}{1+10^{\left(logEC_{50}-x\right)\cdot Hillslope}}$$

### 3.5. Saturation Transfer Difference NMR

Saturation transfer difference NMR was performed on a Bruker AVANCE III 600 MHz (^1^H) NMR spectrometer with a QCI cryoprobe at 25 °C. Test compounds (to 5 mM) and Slt35 (to 20 μM) were added to 50 mM potassium phosphate (450 μL final volume) with 50 μL of D_2_O. The spectra were produced using excitation sculpting for solvent suppression, a relaxation delay of 6 s and a saturation time of 5.9 s containing a train of 50 millisecond E-BURP shaped pulses at 0.5 ppm. For each experiment a corresponding control spectrum was taken whereby the saturation was performed in the absence of the enzyme; no signal enhancement was observed in this case. 

### 3.6. Determination of K_d_

Dissociation constants were determined by fluorimetric titration, using an LS-22 Luminescence Spectrometer (Perkin-Elmer, Buckinghamshire, UK) attached to a Julabo F25 water bath (Julabo, Seelbach, Germany). A total volume of 2 mL of 3.0 μM enzyme in the same buffer as that used for the turbidometry assay was titrated by increasing the concentration of inhibitors. Fluorescence spectra of the enzyme and enzyme–inhibitor solution was recorded after each addition. Three independent repeats were performed for each inhibitor. The excitation wavelength and slit width were 280 and 10 nm respectively, and the emission wavelength and slit width were 290–450 and 5 nm respectively. The maximum emission of lysozyme was at 348 nm while that of Slt35 was at 344 nm. The fluorescence intensity at maximum wavelength was corrected for dilution and for a small inner-filter effect, and converted to a fluorescence difference:

(4) $$∆I_{f}=I_{f}^{initial}-I_{f}^{final}$$

The fluorescence difference data were then fitted by nonlinear regression using SigmaPlot 10 (Ligand Binding, one site saturation):

(5) $$∆I_{f}=\frac{B_{max}\cdot x}{K_{d}+x}$$

### 3.7. Antibacterial Activity

An overnight culture of *E. coli* JM109 in the LB medium was diluted to an OD_500_ of 0.5 and 100 μL aliquots that were spread onto fresh LB-agar (2% agar) plates. Ampicillin and thionine acetate were dissolved in water to give 3 mg/mL and 100 mg/mL solutions respectively. Aliquots of these solutions, to give 30 μg ampicillin and 125–1000 μg thionine acetate, were applied to 5 mm diameter filter paper discs, which were placed onto the surface of the agar. Plates were incubated at 37 °C for 24 h and inhibition zones observed. Each compound was tested in triplicate. 

## 4. Conclusions

A range of glycosidase inhibitors were found to have no inhibitory activity against either the hen egg white lysozyme or Slt35, whereas thionine acetate exhibited a high micromolar inhibition of both enzymes. Although the structure of thionine does not mimic the structure of peptidoglycan enzymes substrate, molecular docking analysis suggested that it is able to block the active sites of both enzymes. The activity of Slt35 was much more sensitive to environmental conditions such as the salt concentration, pH and temperature than that of the lysozyme and Slt35 was inhibited by high concentrations of the substrate while lysozyme was not. However, fluorescence results revealed strong binding affinities of thionine to both enzymes and inhibition constants were similar for the two enzymes. This work could provide groundwork towards the design of effective inhibitors for LTs based on modifications to the thionine structure.

## Figures and Tables

**Table 1:** Effect of thionine acetate on the inhibition zone diameter of ampicillin against *E. coli*.

Mass of Thionine Acetate/μg	Inhibition Zone Diameter/mm
0	25 ± 1
125	25 ± 2
250	28 ± 1
500	30 ± 1
1000	31 ± 1

## Data Availability

Data are contained within the article or Supplementary Materials.

## Supplementary Materials

The following are available online, Figure S1. Saturation transfer difference NMR spectrum (A) and solvent-suppressed 1D 1H NMR spectrum (B) of 20 μM Slt35 and 5 mM 1-deoxynojirimycin, in 50 mM potassium phosphate buffer (pH 7.0) at 25 ºC. Signals at 7–8 ppm in B are from residual imidazole; these signals are not visible in the saturation transfer difference spectrum (A). Figure S2. Comparison of the predicted binding mode of thionine with hen egg white lysozyme (PDB 2VB1) according to BUDE (thionine shown as orange sticks), and thionine manually docked into hen egg white lysozyme (PDB 6LYZ) based on the results of Shanmugaraj et al. (thionine shown as blue-green sticks). The enzyme is shown in pink as a surface representation of the binding site (left), and a cartoon representation showing the amino acid residues (as sticks) located in the active site close to the inhibitor (right). The catalytic Glu35 and Asp52 residues are shown with the backbone ribbon coloured yellow. The predicted hydrogen bonding interactions are shown as green dashes with distances in Å. Figure S3. Comparison of the predicted binding modes of thionine with hen egg white lysozyme (PDB 2VB1) according to BUDE (thionine shown as orange sticks) and AutoDock (thionine shown as green sticks). The enzyme is shown in pink as a surface representation (left) or as a cartoon representation with key active-site residues shown as sticks (right). The catalytic Glu35 and Asp52 residues are shown with the backbone ribbon coloured yellow. Enzyme-ligand hydrogen bonds are shown in green with the distances indicated. Figure S4. Comparison of the predicted binding mode of thionine with hen egg white lysozyme (PDB 6LYZ) according to AutoDock (thionine shown as orange sticks), and thionine manually docked into hen egg white lysozyme (PDB 6LYZ) based on the results of Shanmugaraj et al. (thionine shown as blue-green sticks). The enzyme is shown in pink as a surface representation (left) or as a cartoon representation with key active-site residues shown as sticks (right). The catalytic Glu35 and Asp52 residues are shown with the backbone ribbon coloured yellow. Enzymeligand hydrogen bonds are shown in green with the distances indicated. Figure S5. Comparison of the predicted binding modes of thionine with Slt35 (PDB 1QUS) according to BUDE (thionine shown as orange sticks) and AutoDock (thionine shown as green sticks). The enzyme is shown in blue as a surface representation (left) or as a cartoon representation with key active-site residues shown as sticks (right). The catalytic Glu162 residue is shown with the backbone ribbon coloured yellow. Enzyme-ligand hydrogen bonds are shown in green with the distances indicated. Figure S6. Comparison of the predicted binding mode of thionine (orange sticks) with Slt35 (PDB 1QUS) according to BUDE, and the experimental crystal structure of Slt35 with bulgecin A (grey sticks) bound (PDB 1D0L). The enzyme is shown in blue as a surface representation (left) or as a cartoon representation with key active-site residues shown as sticks (right). The catalytic Glu162 residue is shown with the backbone ribbon coloured yellow.

## Sample Availability

The plasmid containing the *slt35* gene is available from the authors.
