# Supplementary material for: The Lysozyme Inhibitor Thionine Acetate Is Also an Inhibitor of the Soluble Lytic Transglycosylase Slt35 from Escherichia coli
Source: Molecules. 2021 Jul 9;26(14):4189. doi: 10.3390/molecules26144189 (PMC8307938; doi:10.3390/molecules26144189)
Supplement: Supplementary file 1 [file molecules-26-04189-s001.zip › molecules-1279085-supplementary.pdf]

Supplementary Information for

# The lysozyme inhibitor thionine acetate is also an inhibitor of the soluble lytic transglycosylase Slt35 from *Escherichia coli*

**Aysha B. Mezoughi [1], Chiara M. Costanzo [2], Gregor M. Parker [1], Enas M. Behiry [1], Alan Scott [1], Andrew C. Wood [1], Sarah E. Adams [1], Richard B. Sessions [3] and E. Joel Loveridge [1,2,*]**

[1] School of Chemistry, Cardiff University, Park Place, Cardiff, CF10 3AT, UK; abamezoughi2013@gmail.com (A.B.M.), gregor.m.parker@gmail.com (G.M.P.), scotta4@cardiff.ac.uk (A.S.), behiryem@cardiff.ac.uk (E.M.B.), andrew.wood@catsci.com (A.C.W.), sarah.adams@evotec.com (S.E.A.)

[2] Department of Chemistry, Swansea University, Singleton Park, Swansea, SA2 8PP, UK; 993910@swansea.ac.uk (C.M.C.), e.j.loveridge@swansea.ac.uk (E.J.L.)

[3] School of Biochemistry, University of Bristol, University Walk, Bristol, BS8 1TD, UK; R.Sessions@bristol.ac.uk

* Correspondence: e.j.loveridge@swansea.ac.uk

Figure S1. Saturation transfer difference NMR spectrum and solvent-suppressed 1D $^1$H NMR spectrum of 20 μM Slt35 and 5 mM 1-deoxynojirimycin, in 50 mM potassium phosphate buffer (pH 7.0) at 25 ºC.

Figure S2: Comparison of the predicted binding mode of thionine with hen egg white lysozyme (PDB 2VB1) according to BUDE, and thionine manually docked into hen egg white lysozyme (PDB 6LYZ) based on the results of Shanmugaraj *et al*.

Figure S3: Comparison of the predicted binding modes of thionine with hen egg white lysozyme (PDB 2VB1) according to BUDE and AutoDock.

Figure S4: Comparison of the predicted binding mode of thionine with hen egg white lysozyme (PDB 6LYZ) according to AutoDock, and thionine manually docked into hen egg white lysozyme (PDB 6LYZ) based on the results of Shanmugaraj *et al*.

Figure S5: Comparison of the predicted binding modes of thionine with Slt35 (PDB 1QUS) according to BUDE and AutoDock.

Figure S6: Comparison of the predicted binding mode of thionine with Slt35 (PDB 1QUS) according to BUDE, and the experimental crystal structure of Slt35 with bulgecin A bound (PDB 1D0L).

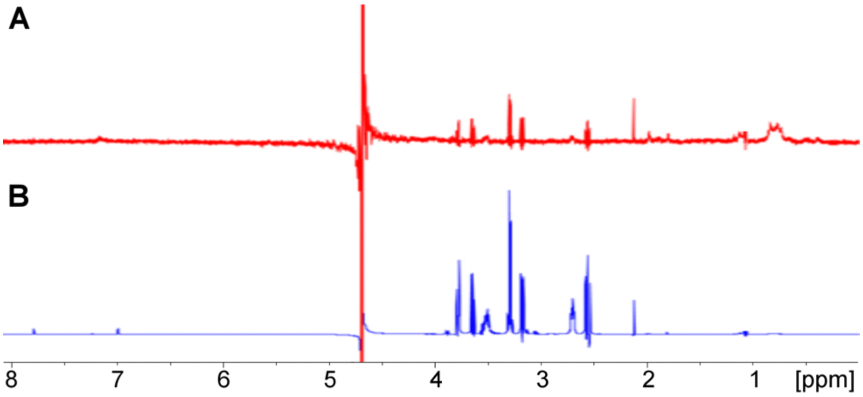


**Figure S1.** Saturation transfer difference NMR spectrum (A) and solvent-suppressed 1D $^{1}H$ NMR spectrum (B) of 20 µM Slt35 and 5 mM 1-deoxynojirimycin, in 50 mM potassium phosphate buffer (pH 7.0) at 25 ºC. Signals at 7-8 ppm in B are from residual imidazole; these signals are not visible in the saturation transfer difference spectrum (A).

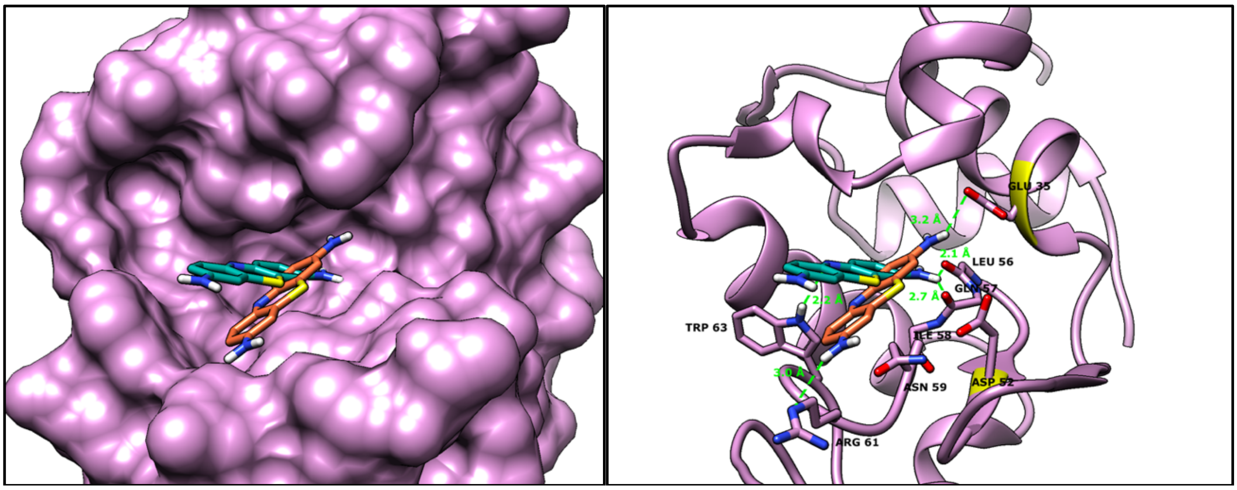


**Figure S2.** Comparison of the predicted binding mode of thionine with hen egg white lysozyme (PDB 2VB1) according to BUDE (thionine shown as orange sticks), and thionine manually docked into hen egg white lysozyme (PDB 6LYZ) based on the results of Shanmugaraj *et al*. (thionine shown as blue-green sticks). The enzyme is shown in pink as a surface representation of the binding site (left), and a cartoon representation showing the amino acid residues (as sticks) located in the active site close to the inhibitor (right). The catalytic Glu35 and Asp52 residues are shown with the backbone ribbon coloured yellow. The predicted hydrogen bonding interactions are shown as green dashes with distances in Å.

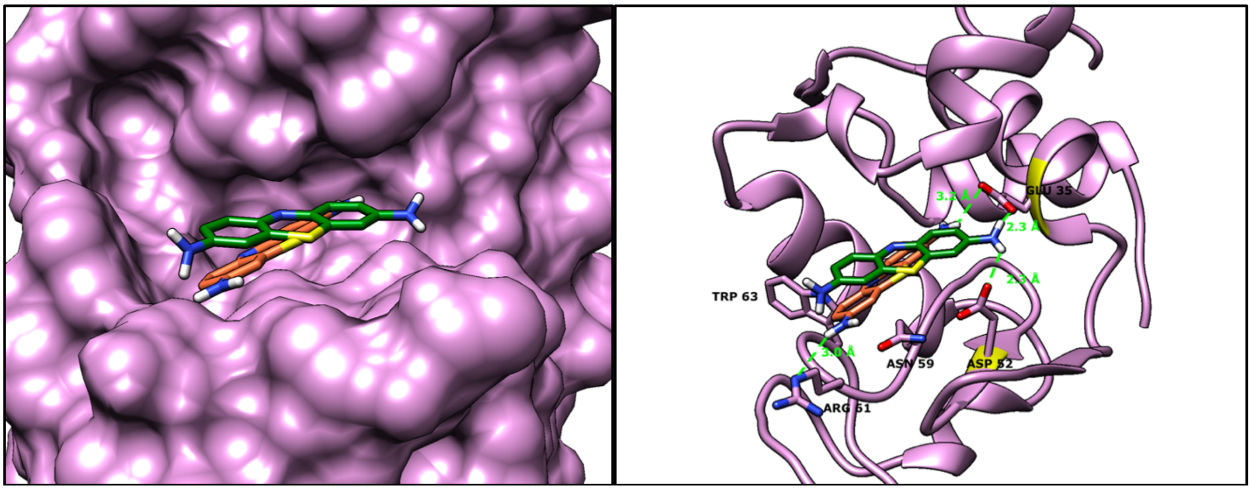


**Figure S3.** Comparison of the predicted binding modes of thionine with hen egg white lysozyme (PDB 2VB1) according to BUDE (thionine shown as orange sticks) and AutoDock (thionine shown as green sticks). The enzyme is shown in pink as a surface representation (left) or as a cartoon representation with key active-site residues shown as sticks (right). The catalytic Glu35 and Asp52 residues are shown with the backbone ribbon coloured yellow. Enzyme-ligand hydrogen bonds are shown in green with the distances indicated.

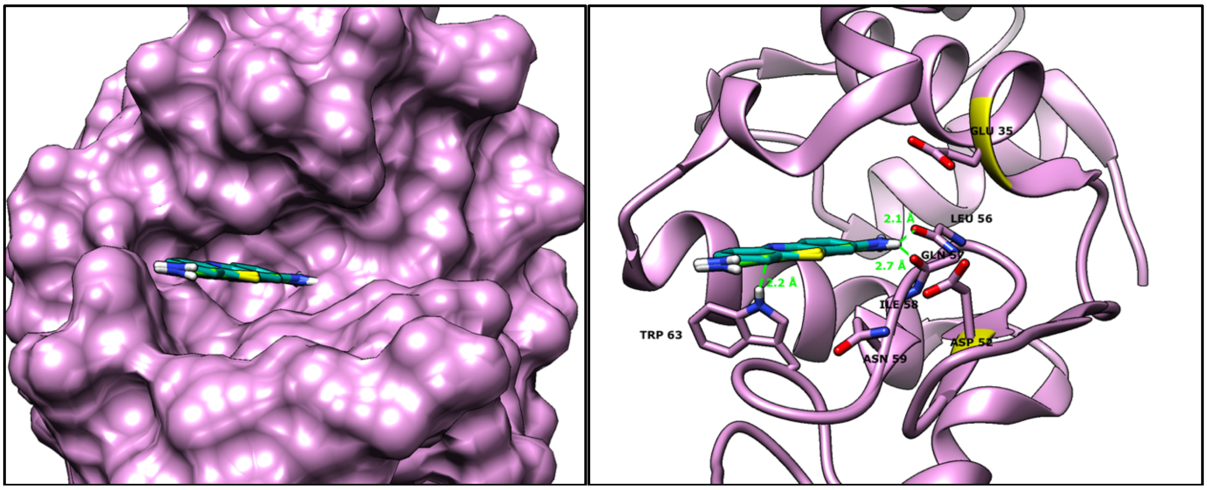


**Figure S4.** Comparison of the predicted binding mode of thionine with hen egg white lysozyme (PDB 6LYZ) according to AutoDock (thionine shown as orange sticks), and thionine manually docked into hen egg white lysozyme (PDB 6LYZ) based on the results of Shanmugaraj *et al*. (thionine shown as blue-green sticks). The enzyme is shown in pink as a surface representation (left) or as a cartoon representation with key active-site residues shown as sticks (right). The catalytic Glu35 and Asp52 residues are shown with the backbone ribbon coloured yellow. Enzyme-ligand hydrogen bonds are shown in green with the distances indicated.

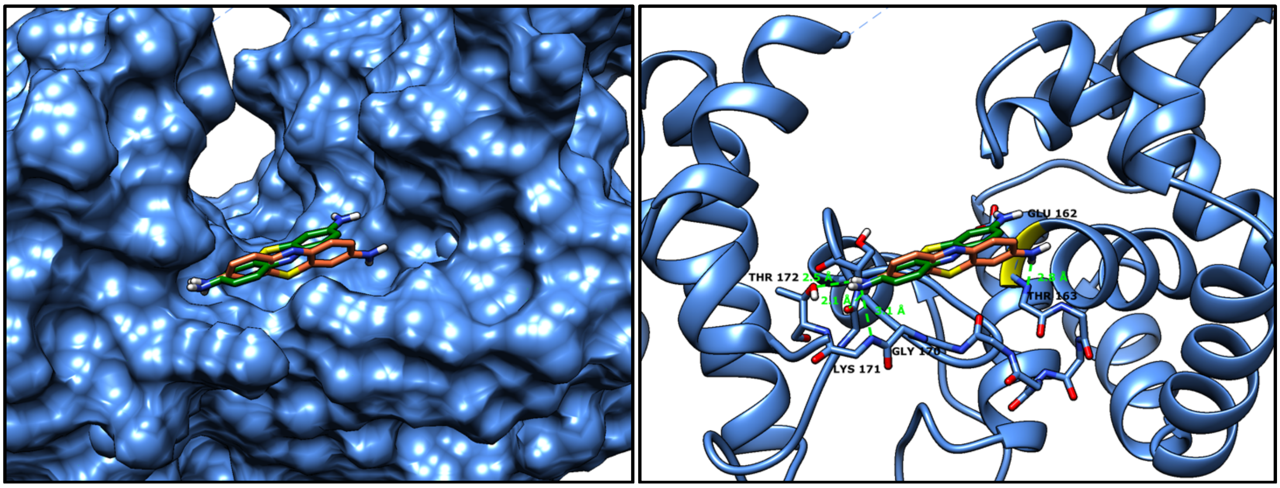


**Figure S5.** Comparison of the predicted binding modes of thionine with Slt35 (PDB 1QUS) according to BUDE (thionine shown as orange sticks) and AutoDock (thionine shown as green sticks). The enzyme is shown in blue as a surface representation (left) or as a cartoon representation with key active-site residues shown as sticks (right). The catalytic Glu162 residue is shown with the backbone ribbon coloured yellow. Enzyme-ligand hydrogen bonds are shown in green with the distances indicated.

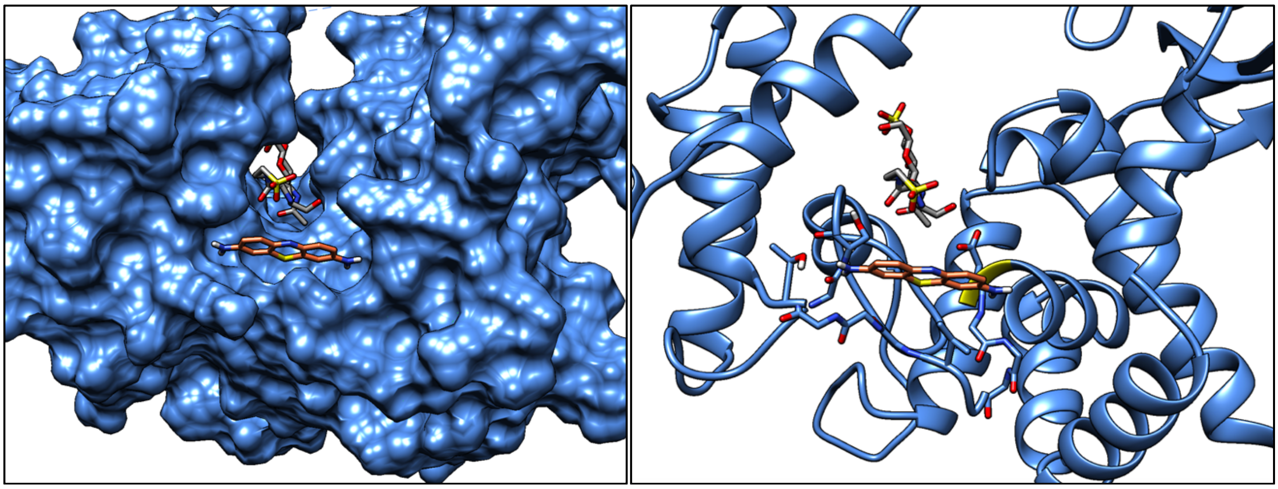

**Figure S6.** Comparison of the predicted binding mode of thionine (orange sticks) with Slt35 (PDB 1QUS) according to BUDE, and the experimental crystal structure of Slt35 with bulgecin A (grey sticks) bound (PDB 1D0L). The enzyme is shown in blue as a surface representation (left) or as a cartoon representation with key active-site residues shown as sticks (right). The catalytic Glu162 residue is shown with the backbone ribbon coloured yellow.
